# Retraction Note: Brief literature review and comprehensive bioinformatics analytics unravel the potential mechanism of Curcumin in the treatment of periodontitis

**DOI:** 10.1186/s12903-025-05744-6

**Published:** 2025-03-15

**Authors:** Xufeng Huang, Ying Liu, Qi Wang, Hafiz Muzzammel Rehman, Dorottya Horváth, Shujing Zhou, Rao Fu, Ling Zhang, Attila Gábor Szöllősi, Zhengrui Li

**Affiliations:** 1 Faculty of Dentistry, University of Debrecen, Debrecen, Hungary; 2 Department of Immunology, University of Debrecen, Debrecen, Hungary; 3 Department of Cardiology, Sixth Medical Center, PLA General Hospital, Beijing, China; 4 Department of Gastroenterology, Affiliated Hospital of Jiangsu University, Jiangsu University, Zhenjiang, China; 5 School of Biochemistry and Biotechnology, University of the Punjab, LahorePunjab, 54590 Pakistan; 6 Alnoorians Group of Institutes, 55-Elahi Bukhsh Park, Amir Road, Shad Bagh, Lahore, 54000 Pakistan; 7 Department of Oral and Maxillofacial-Head and Neck Oncology, College of Stomatology, Shanghai Ninth People’s Hospital, Shanghai Jiao Tong University School of Medicine, Shanghai Jiao Tong University, Shanghai, China; 8 National Center for Stomatology and National Clinical Research Center for Oral Diseases, Shanghai, China; 9 Shanghai Key Laboratory of Stomatology, Shanghai, China

**Retraction Note: BMC Oral Health (2023) 23:469**

10.1186/s12903-023-03181-x

The Editor has retracted this article after concerns were raised about the data reported. The two datasets used in this analysis, GSE10334 and GSE16134, were each generated from the same archive of healthy and diseased gingival tissues, and as such there is a significant sampling overlap between the two. Furthermore, both datasets contain multiple samples per patient. The analytical approach employed by the authors in this paper does not account for these limitations. The Editor no longer has confidence in the reliability of the article’s results and conclusions.

Zhengrui Li, Attila Gábor Szöllősi, Hafiz Muzzammel Rehman, Xufeng Huang, Qi Wang, Shujing Zhou, and Rao Fu disagree with this retraction. The remaining authors did not respond to correspondence from the Publisher regarding this retraction.

